# Five Skills Medical Students Should Have

**DOI:** 10.31729/jnma.4878

**Published:** 2020-04-30

**Authors:** Mandeep Guragai, Deependra Mandal

**Affiliations:** 1 Kathmandu Medical College and Teaching Hospital, Sinamangal, Kathmandu, Nepal

## INTRODUCTION

A doctor is not a software filled with pages of codes that identifies symptoms, makes a diagnosis, and formulates a management plan for patients. A doctor is a human being who has to not just access the vast volumes of information he/she has learned but also has to maintain a connection with the patients while treating them. For this, medical schools must follow strategies to enhance and improve qualities in medical students other than academics. A professor of medical education mentions that the students must be provided with an environment of learning by doing and appropriate clinical exposures at different phases of training.^1^ This is exemplified by the fact that during our training, we are required to interact with patients, ask about their difficulties regarding health and learn to empathize.

There is a defined term in literature known as non-technical skills (NTS) that are the cognitive, social and personal resource skills; recognized in healthcare as being crucial to the provision of high-quality, safe and effective patient care.^2^ Examples of such would be the skills of decision making, communication, and teamwork.^3^

The question of what makes a good doctor is not easy to answer. But, there are some skills that doctors must have and must acquire through their days of training to become a better physician. An attempt to list out all the skills required would be extremely difficult. Hence, we have listed five skills that medical students should have, in no particular order.

## SKILL 1: ACADEMIC COMPETENCE

There is no doubt that students need to have a good knowledge of medical science. For a practicing doctor, a strong knowledge base is required to provide the best possible care to the patients. Which is why medical students spend a large chunk of time in studying. We learn about the etiology, pathophysiology, diagnosis, and treatments for diseases in medical school. We also have to learn to take a good history, find a suitable diagnosis and at least know how the disease would be treated.

## SKILL 2: COMMUNICATION

Good communication skill is a valuable asset for students and doctors. It helps doctors identify patients' problems distinctly and patients have greater adherence to therapy.^4^ While the advantages of good communication skills are not limited to a better doctor- patient relationship. As future healthcare professionals, very rarely would we be working alone or individually on something. Rather, we would always be in a team, be it single-disciplinary or multidisciplinary. Proper communication is always required when working with a team.

There is some evidence to suggest that the students' communication skills deteriorate during the clinical years.^5^ While we spend the majority of our time acquiring medical knowledge and skills, investing some time to improve our communication skills can provide the ‘humane’ touch to our future clinical practice. Communication can also help address the elephant in the room. Many-a-times miscommunication or not communicating leads to conflicts, disputes, disagreements among peers and colleagues.

The work culture at the Journal of Nepal Medical Association (JNMA) is such that we are encouraged to express ourselves and periodically give and receive feedback. This, being a part of effective communication, has led to the formation of a highly efficient and well-bonded workforce of assistant editors and editorial support members.

## SKILL 3: LEADERSHIP

As doctors, we will be faced with numerous situations where the type of decision we make can drastically change the outcome for someone's health. Not just patient-related decisions, doctors are sometimes required to make administrative decisions (for example, as a hospital director, head of the department, etc). Doctors being appointed to hospital boards of directors has shown to be positive for clinical outcomes and overall performances.^6^

We will always work in a group, be it while taking a history of a patient, or while managing a case, and so on. A group requires a leader who has to set in motion its activities. Doctors even organize health camps, seminars, workshops, conferences, etc. where the process of organizing these events would require leadership skills from us.

Leadership is an essential skill for medical students, but not addressed by our curriculum in Nepal. A qualitative study done in a medical school in the UK reports that students suggested topics like team working skills, decision-making and negotiating skills, to be integrated into the training to make leadership and management education relevant in the clinical context.^7^

## SKILL 4: TEAMWORK

Working as a part of a team is a skill. Whenever working with a group, it is important to understand that all members have their unique personalities and perspectives. These differences, if not managed, could be the barriers to the functioning of the team. A relatable example for medical students would be when a few of them go to take a history of a patient, and all are enthusiastic about asking him/her questions. If the team doesn't appoint or allow a single person to take a history, multiple questions from multiple people might irritate the patient, or allow irrelevant or out-of-context questions to be asked. This would make for an incoherent and unproductive history-taking session.

A team must identify every member's strengths and weaknesses, and then identify the objective of the team. If the objective is to get the work done as efficiently as possible, work should be divided according to the strength of each individual. But if the objective is for the team members to learn or grow, they must be given a chance to work on their weaknesses. Therefore, contextually the team must give each other their space while also working together.

A study has found that teamwork has a medium-sized effect on performance and also recommends healthcare organizations to emphasize approaches that maintain and improve teamwork.^8^

## SKILL 5: EMPATHY

While interacting with patients in the wards, or while practicing a new clinical skill on a patient, we often forget that we might be causing the patient unnecessary discomfort and continue to do so. Although we enquire about the symptoms the patient is experiencing, we cannot fully experience their suffering. Therefore, empathizing with a patient is rather an intellectual skill rather than an emotional form of knowing.^9^

Empathy is the capacity to understand another person's experience from within that person's frame of reference.^10^ Simply asking the patients to elaborate more on the feelings that they might be having or asking them how a particular disease or symptom is affecting them can be a form of empathizing with them. Acknowledging that they are in pain, or are irritated/frustrated can build a better rapport. Derksen et al. concluded in their systematic review that due to a good correlation between physician empathy and patient satisfaction, its importance in general practice is unquestionable.^11^

## WAY FORWARD

A medical student must learn much more than just what is written in books. Each skill could take a lot of practice to develop, but acquiring only one or some of these skills might also not be enough. These skills, with many others, are to a great extent interrelated and might develop at the same time. Just like leading a team would teach communication skills, leadership skills, teamwork and much more. One must remember that the ultimate goal of learning these skills is the best healthcare delivery to our patients of the future.

## Conflict of Interest

**None.**
